# A Home Exercise Programme Is No More Beneficial than Advice and Education for People with Neurogenic Claudication: Results from a Randomised Controlled Trial

**DOI:** 10.1371/journal.pone.0072878

**Published:** 2013-09-30

**Authors:** Christine Comer, Anthony C. Redmond, Howard A. Bird, Elizabeth M. A. Hensor, Philip G. Conaghan

**Affiliations:** 1 Leeds Musculoskeletal and Rehabilitation Service, Leeds Community Healthcare, Leeds, United Kingdom; 2 Leeds Institute of Rheumatic and Musculoskeletal Disease, Faculty of Health, University of Leeds, Leeds, United Kingdom; 3 NIHR Leeds Musculoskeletal Biomedical Research Unit, Leeds Teaching Hospitals National Health Service Trust, Leeds, United Kingdom; The James Cook University Hospital, United Kingdom

## Abstract

**Objective:**

To compare the effectiveness of a physiotherapy programme with a control treatment of advice and education in patients with neurogenic claudication symptoms.

**Design:**

Pragmatic randomised controlled clinical trial.

**Setting:**

Primary care-based musculoskeletal service.

**Patients:**

Adults aged 50 or over with neurogenic claudication symptoms causing limitation of walking.

**Interventions:**

Condition-specific home exercises combined with advice and education, or advice and education alone.

**Main outcome measures:**

The primary outcome was the difference in improvement of symptom severity scores on the Swiss Spinal Stenosis Scale at eight weeks. Secondary outcomes included measures of physical function, pain and general well-being at eight weeks and 12 months.

**Results:**

There was no significant difference between groups in the Swiss Spinal Stenosis symptom severity scale at eight weeks (t = 0.47, p = 0.643): mean change (SD) control group −0.18 (0.47), treatment group −0.10 (0.66), difference (95% CI) 0.08 (−0.19, 0.35); baseline-adjusted difference 0.06 (−0.19, 0.31)]. An unplanned subgroup analysis suggested that for patients with the top 25% of baseline symptom severity scores, the physiotherapy exercise programme resulted in an improvement in the primary outcome, and modest but consistently better secondary outcomes at both time-points compared to the control group. The effectiveness in different subgroups requires further direct evaluation.

**Conclusions:**

In the treatment of patients with neurogenic claudication symptoms, a physiotherapist-prescribed home exercise programme is no more effective than advice and education.

**Ethical approval:**

The study was approved by *Leeds Central Ethics Committee and informed consent was given by all participating patients.*

**Copyright:**

The Corresponding Author has the right to grant on behalf of all authors and does grant on behalf of all authors, a worldwide licence to the Publishers and its licensees in perpetuity, in all forms, formats and media (whether known now or created in the future), to i) publish, reproduce, distribute, display and store the Contribution, ii) translate the Contribution into other languages, create adaptations, reprints, include within collections and create summaries, extracts and/or, abstracts of the Contribution, iii) create any other derivative work(s) based on the Contribution, iv) to exploit all subsidiary rights in the Contribution, v) the inclusion of electronic links from the Contribution to third party material where-ever it may be located; and, vi) licence any third party to do any or all of the above.

**Trial registration:**

ISRCTN 78288224 – doi10.1186/ISRCTN35836727; UKCRN 4814.

## Introduction

Patients with lumbar spinal stenosis (LSS) classically present with symptoms of neurogenic claudication (NC); these symptoms are described as leg pain, numbness and heaviness brought on by walking and relieved when the spine is flexed, for example when stooping or sitting [1]. The symptoms of NC can cause significant limitations in walking, requiring patients to seek treatment for their symptoms [2]. Lumbar spinal stenosis is the most common reason for spinal surgery in patients over the age of 65, but as surgical outcomes are variable, conservative treatment is generally recommended in the first instance and the majority of patients are therefore referred for assessment and treatment by physiotherapists at some point in the course of the condition.

When patients with NC symptoms are referred for physiotherapy treatment, they are commonly prescribed home exercise programmes to include spinal flexion and stabilisation exercises in addition to aerobic fitness exercises [3]. These exercise choices reflect recommended programmes which are based on the theoretical benefits of modifying posture to reduce the lordotic curve and minimise the extension forces through the lumbar spine and thereby optimising the available space for the spinal nerves [4]–[7]. There is, however, little evidence from clinical trials regarding effectiveness. It has been shown that lumbar posture can be modified with exercises [8] and the few clinical trials of LSS which have included exercise therapy as part of a package of conservative treatments, suggest that exercise therapy consisting of flexion-based spinal movements, lumbopelvic stabilisation and posterior pelvic tilting exercises may be beneficial [9]–[11]. The clinical effectiveness of such condition-specific exercise programmes when used as a primary care intervention has not, to-date, been evaluated adequately.

The effects of exercise therapy on function and symptoms may not be expected to match those for surgical interventions, but it is known that the longer term results from surgery tend towards deterioration and there are obvious inherent risks associated with surgical treatments. It remains important, therefore, to establish whether conservative treatments such as physiotherapy exercises can offer an acceptable alternative in the management of NC. The aim of this trial was to evaluate the effectiveness of a condition-specific home exercise programme, focusing on posture modification and aerobic fitness. Specifically, the trial was designed to compare outcomes in measures of pain and function in people with NC receiving a typical six-week, physiotherapist prescribed home exercise programme, compared to a control group receiving advice and education alone.

## Methods

The study was submitted for review through IRAS, and was approved by the Leeds Central Ethics Committee. A two-arm randomised controlled trial design was used. The protocol for this trial and supporting CONSORT checklist are available as supporting information; see CONSORT checklist S1 and Protocol S1. In this pragmatic trial, patients were recruited from general practitioner referrals to the Leeds Musculoskeletal and Rehabilitation Service, a primary care-based musculoskeletal service. Inclusion and exclusion criteria for recruitment to the study are presented in Table 1. Severe cases of spinal stenosis (those with acute cauda equina syndrome or worsening neurological status) who were likely surgical candidates were excluded. Patients recruited to the trial were therefore typical of patients with mild to moderate LSS, who are commonly referred to primary care services for physiotherapy treatment in the first instance. MRI confirmation of lumbar spinal stenosis was not an inclusion criterion for this study, as it was intended that the trial should focus on the evaluation of a treatment for the clinical syndrome of NC as currently recognised and treated by physiotherapists in the primary care setting. Patients were included therefore on the basis of symptoms on clinical assessment that were consistent with NC and which would have entered them onto a primary care physiotherapy intervention pathway in normal NHS practice.

**Table 1:** Inclusion and exclusion criteria.

Inclusion criteria	Exclusion criteria
Age 50 years or over	Cognitive impairment or other medical conditions preventing understanding or participation in the study
Bilateral neurogenic claudication symptoms (ie exercise induced leg pain on walking, relieved in sitting or flexion)	Clearly defined radicular symptoms (ie single nerve root symptoms)
Patient-reported limitation in walking tolerance due to NC symptoms	Signs or symptoms of acute cauda equina syndrome or severe or worsening neurological status requiring medical or surgical assessment. (This includes significant or worsening nerve root or cauda equina function, significant or sinister weight loss, pyrexia, unremitting pain, significant inflammatory joint disease)

After screening for eligibility, potential participants provided informed written consent, and were then randomised to the relevant treatment arm determined by random permuted block randomisation (block sizes 2, 4 or 6) without stratification via a commercial web-based computer-generated randomisation protocol. The block size was not revealed to the trial co-ordinator or treating physiotherapists, and a sealed envelope system was used by administrative support staff to conceal treatment allocation from participants and physiotherapists until the first treatment appointment. The trial co-ordinator remained blinded to the treatment allocation until all final follow-up data was received and collated. Statistical analysis was undertaken with the statistician blind to treatment allocation.

### Interventions

All interventions were delivered by a team of 28 senior musculoskeletal physiotherapists working in the primary care setting, who received specific training and a written manual before the start of the trial. Participants randomised to the control group received advice and education provided in both verbal and written format (see Appendix S1) at the initial physiotherapy appointment and were given a contact telephone number of the treating physiotherapist for further contact and advice if needed during the six week treatment period. Participants randomised to the active treatment group received the same standardised advice and education as the control group and in addition, they were prescribed a condition-specific home exercise programme to be carried out twice daily at home over a six week period. The set of exercises was selected to reflect a combination of current physiotherapy practice as evidenced by recent practitioner survey data [3], [12] and recommendations in the available literature [4]–[7]. The constituent exercises focussed on 1) flattening of the lumbar lordosis 2) lumbar flexion 3) abdominal muscle activation 4) trunk muscle strengthening and 5) aerobic fitness (see Appendix S2). Participants were taught how to perform one of each of these five types of exercise at their first physiotherapy appointment, and were then instructed to perform the exercises at least twice daily at home. Exercise technique, difficulty levels and number of repetitions of each exercise were reviewed and progressed at subsequent physiotherapy appointments, and adherence to the home exercise programme throughout and after the six week treatment period was encouraged by the treating physiotherapist. As the structure of these interventions aimed to reflect typical primary care management in current clinical practice, which would generally consist of the provision of appropriate advice, and the prescription of home exercises for self-management, compliance was not formally evaluated. Any additional treatments received during the trial period (e.g. walking aids, spinal injections) were also documented.

### Outcome measures

The primary measure of outcome was the change in the symptom severity subscale score of the Swiss Spinal Stenosis (SSS) scale [12] at eight weeks (two weeks after completion of the six week treatment period). The symptom severity scale was felt to best reflect changes important to patients with neurogenic claudication symptoms seeking medical care. Secondary endpoints included 8 week changes in the physical function subscale of the SSS, the General Well-Being Index (GWBI) [13], Oswestry disability questionnaire [14], and a visual analogue scale for back pain and leg pain. The Hospital Anxiety and Depression Scale (HADS) [15] was collected at baseline to investigate the potential impact of psychological distress on improvements in patient-reported pain and quality of life. All data for these outcomes were collected via questionnaires completed by patients either in clinic or at home, without guidance from the treating physiotherapist. In addition, walking tolerance was measured using a shuttle walking test (SWT), which has been shown to be a reliable and responsive measure in patients receiving treatment for chronic spinal problems, including LSS, and to correlate well with self-reported functional walking items in outcome measures such as the EQ5D and SF36 [16]–[18]. Whilst the SWT may not give a true reflection of walking capacity in a patient’s normal environment, it is easy to use in the clinical setting, requiring patients to walk up and down a 10 metre course at increasing speed for each minute, dictated by signals from a pre-recorded audiotape, up to a maximum of 12 minutes (1020 metres). Evaluation of longer term outcomes was based on postal questionnaire follow-up to measure changes at 12 months in all measures other than the shuttle walking test.

### Sample size

The required sample size (n = 76) was determined *a priori*, based on the ability to detect a difference (Δ) between the treatment groups equivalent to the previously determined minimum clinically important difference (MCID) of 0.5 points for the SSS symptom severity scale [19], and assuming a standard deviation of 0.56 [20], [21] with power set at 90% (alpha 5%), and allowing for a drop-out rate of 20%.

### Rasch-transformation of questionnaire data

To provide interval scaling, the ordinal data from each of the questionnaires was transformed to interval scaling via Rasch analysis using RUMM2030. All scales demonstrated adequate fit to the Rasch model and strict unidimensionality [22] [SSS (Chi-square 4.28, df 10, p = 0.934; 4.88% of independent t-tests significant); Oswestry (Chi-square 23.92, df 14, p = 0.091; 2.96% of independent t-tests significant); GWBI (Chi-square 2.81, df 4, p = 0.590; 3.83% of independent t-tests significant)].

### Statistical tests

Patients with data available at each endpoint were included in the analyses according to their original treatment allocation. Multiple imputation by chained equations was performed to account for missing data; 20 imputed datasets were generated. The imputation model included baseline data for all efficacy variables in addition to exploratory confounders and auxiliary variables found to be associated with the values of the outcome at Pearson’s |r|≥0.5. Binary logistic regression models of the probability that data were missing at follow-up were created; variables found to be associated with missingness at p<0.1 were also included as auxiliary variables. Imputation was performed in each treatment group separately to allow for interaction effects to be investigated. Missing baseline data were handled using mean imputation which has been recommended for randomised trials where there is a need to limit the number of covariates in the imputation model [23]. All efficacy outcomes were imputed using predictive mean matching. Changes in the primary outcome measure (SSS symptom severity score) and secondary and exploratory outcome measures were computed passively and were initially explored descriptively in each group. Subsequent inferential analysis of these data used linear regression; each model included an indicator variable denoting treatment, and the baseline values of the dependent variable. Preliminary checks were conducted to ensure that linear regression assumptions of normality and homoscedasticity of residuals were not violated. Analyses were repeated using robust quantile regression, which does not require errors to be normally distributed or homoskedastic. Both unadjusted and adjusted results are presented. Results from the imputed datasets were combined using Rubin’s rules. Multiple imputation assumes data are missing at random, i.e. the likelihood that the data is missing does not depend on the value of the data that is missing (for example, patients with more severe symptoms being less likely to have complete severity data). As a sensitivity analysis the values imputed were altered to reflect a situation in which patients with missing data had improved or deteriorated, either in both groups simultaneously or in one or the other group, thus assuming the data were missing not at random. Additional sensitivity analyses were undertaken where appropriate controlling for imbalances between the treatment groups in other baseline characteristics. A per protocol analysis was also performed which included patients in the control arm who had received just one physiotherapy session and patients in the active treatment arm who had attended at least 3 physiotherapy sessions; we then extended this to exclude patients who reported having had surgery or injections to the spine during the course of the study. We also performed a complete case analysis which included only those patients with data available at each time-point. Because the use of block randomisation could theoretically permit researchers to subvert the allocation concealment for some patients, we used a variety of methods to identify selection bias in our data. We calculated P(E), the probability of being randomised to the experimental (treatment) arm for each participant, based on knowledge of their position within the randomisation block and knowledge of the groups to which preceding patients in the block had been assigned [24]. To identify observed selection bias we assessed the magnitude of the between-group differences at baseline for patients where P(E)≠0.5. To identify unobserved selection bias, we included P(E) as a covariate in the analysis models. All tests were two-tailed at the 0.05 level of significance; corrections for multiple comparisons were made on a family-wise basis for all secondary endpoint analyses using the Holm method. The threshold for significance at the 5% level was consequently set to p = 0.006 for the secondary endpoints. Exploratory endpoint analyses were not adjusted for multiple comparisons. Analyses were performed in Stata 12.1.

## Results

**Figure 1:**
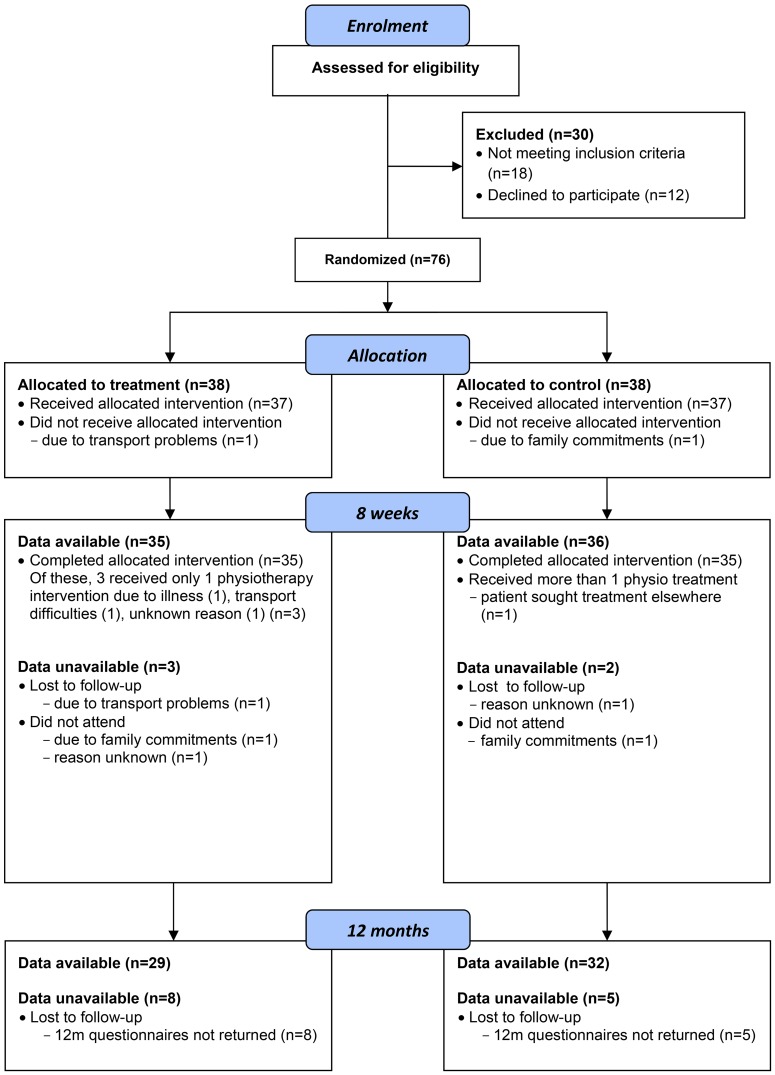
Flow chart of participants through trial.

One hundred and six potential participants were screened over a 17 month enrolment period, resulting in 76 recruits to the trial. Forty were female, with a mean age in the sample of 73 years (SD 8.7 years), a mean baseline symptom severity score of 3.05 (SD 0.61), physical function score 2.36 (SD 0.50) and mean number of 10 metre walking test shuttles 22.15 (SD 18.15). Follow-up data were available for 71 (93%) participants at 8 week follow-up and 61 (80%) participants at 12 months (see flow-chart Figure 1).

### Missing data

The level of missing data at the primary endpoint was below 10% in each group and where data were missing this was generally due to reasons considered to be unrelated to the patients’ health (see flow-chart Figure 1). The pattern of missing data was deemed arbitrary rather than monotone; 3 patients who did not attend for follow-up at 8 weeks nevertheless returned the 12 month questionnaires. Mean imputation was used for missing baseline covariates because the missing indicator method caused colinearity problems with the imputation model; mean imputation has been recommended where there is a need to limit the number of covariates in the imputation model [23]. Age, gender and symptom duration were included in the imputation model because these variables were to be included in sensitivity analyses. No further auxiliary variables were identified (data not shown). Monte Carlo errors for the regression coefficients, t-statistics and p-values were adequate according to published guidelines [25], indicating that 20 imputations were sufficient to achieve stable results.

The baseline characteristics of each group are presented in Table 2. Although MRI confirmation of LSS was not specified as an inclusion criterion, 43 of the 76 participants reported at the time of recruitment that they had undergone a previous MRI scan of the spine and the radiologist reports were traced and reviewed where available. Of the thirty-seven MRI reports which could be obtained (48.7% of all participants), all confirmed the presence of LSS, supporting the clinical diagnosis of this condition by musculoskeletal physiotherapists in the primary care clinical setting. The treatment groups were generally well balanced in most baseline demographic and clinical characteristics, however patients in the control group were on average five years younger, fewer were female, and they had experienced symptoms for longer.

**Table 2:** Baseline characteristics.

	Control n = 38	Active n = 38
Age, years: mean (SD), range	70.8 (8.3), 53 to 87	75.3 (8.6), 54 to 86
Female: n (%)	18 (47.4%)	22 (57.9%)
BMI: mean (SD), range	28.10 (4.34), 20.8 to 37.1 (n = 36)	28.30 (5.32), 22.5 to 49.2 (n = 35)
Duration*, years: median (IQR)	10.0 (2.8 to 35.8)	3.5 (1.0 to 10.0)
Spinal MRI report available	23 (60.5%)	20 (52.6%)
SSS symptom: mean (SD)	3.3 (0.5)	3.2 (0.6)
SSS physical: mean (SD)	2.6 (0.4)	2.2 (0.4)
Shuttles completed: median (IQR)	21.0 (12.0 to 34.5)	15.0 (5.5 to 27.5) (n = 37)
Oswestry score: mean (SD)	43.4 (9.5)	42.1 (7.7)
General Well-Being Index: mean (SD)	65.68 (14.44)	67.26 (11.60) (n = 35)
Back pain VAS: mean (SD)	63.2 (29.4)	55.2 (29.6) (n = 37)
Leg pain VAS: mean (SD)	67.6 (22.8)	64.5 (30.0) (n = 36)
HADS depression: mean (SD)	9.0 (3.2)	9.3 (2.4) (n = 37)
N physio sessions: median (IQR), range	1 (1 to 1), 0 to 2	3 (2 to 3), 0 to 4 (n = 36)

* Time since onset of first symptom (back and/or leg pain).

### Testing for observable selection bias at baseline

Although some differences were observed between the randomised groups at baseline (Table 2), these were not in a consistent direction. Controls were younger, a smaller proportion of the group was female, they had symptoms of longer duration, and they completed more shuttles. There were no substantive differences in any of the patient-reported outcomes and in particular the groups were very well matched in terms of SSS symptom severity (3.3 vs. 3.2). Restricting the analysis to patients in whom P(E)≠0.5 yielded differences of equal or reduced magnitude for the majority of outcomes (Table S1).

### Primary outcome

**Figure 2:**
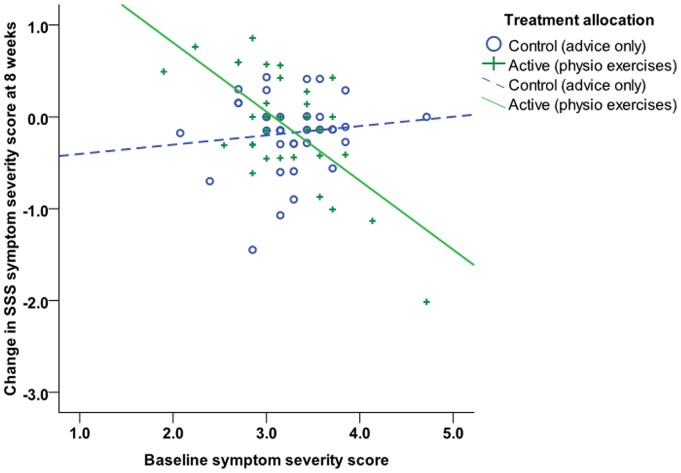
Change in SSS symptom severity score at eight weeks plotted against baseline values in the control group (circles; dotted line) and treatment group (crosses; solid line).

One patient in the active therapy arm performed the shuttle test at week 8 but did not complete any of the questionnaires; therefore change in SSS symptom severity at eight weeks was available for 36 patients in the control arm and 34 in the active treatment arm. Multiple imputation allowed all 38 patients in each group to be included in the analysis. Mean (SD) unadjusted change in SSS symptom severity at eight weeks was −0.18 (0.47) in the control arm and −0.10 (0.66) in the active treatment arm [unadjusted mean (95% CI) between-group difference 0.07 (−0.18, 0.32); baseline-adjusted difference 0.05 (−0.19, 0.29)]. The primary analysis showed no significant difference between the groups (t = 0.42, p = 0.676). Subsequent analysis investigating effect modification indicated that interpretation of the main effects in the adjusted analysis was complicated by an interaction between baseline symptom severity and score changes in the treatment group (t = −3.84, p<0.001), such that participants in the active treatment group with relatively low baseline symptom severity scores deteriorated, and those with higher baseline scores generally improved, whereas for subjects in the control arm, baseline values had little influence over the degree of change observed (Figure 2 shows change in SSS symptom severity score at eight weeks plotted against baseline values in the control group [circles; dotted line] and treatment group [crosses; solid line]).

The efficacy of the treatment appeared, therefore, to vary with the level of pre-treatment symptom severity. A number of unplanned analyses were therefore conducted to control for this interaction. Firstly, the interaction was assumed to be a genuine property of the relationship between treatment and symptom severity and so to help quantify the interaction effect the mean change in each treatment group at different baseline levels of symptom severity was estimated from the regression equation:
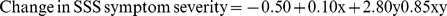
where x is baseline SSS symptom severity, y = 0 for control group, y = 1 for active group.

Estimated changes for the minimum and maximum, upper and lower quartiles and median of the distribution of baseline values are presented in Table 3. The predicted values indicated a between group difference for baseline symptom severity scores at or above the 75^th^ percentile.

**Table 3:** Predicted values of the primary outcome, change in SSS symptom severity score at eight weeks, at varying levels of baseline SSS symptom severity.

	Baseline-adjusted change at 8 weeks		
SSS symptom severityat baseline	Control n = 38	Active n = 38	Difference (95% CI)
**Minimum ( = 1.90)**	−0.31	0.88	1.19 (0.56, 1.81)
**25^th^ percentile ( = 2.96)**	−0.20	0.08	0.29 (0.04, 0.53)
**Mean ( = 3.22)**	−0.18	−0.12	0.06 (−0.15, 0.28)
**75^th^ percentile ( = 3.57)**	−0.14	−0.38	−0.23 (−0.50, 0.03)
**Maximum ( = 4.71)**	0.03	−1.23	−1.20 (−1.89, −0.51)

Secondly, it was assumed that the interaction between treatment and symptom severity may be a manifestation of regression to the mean, which was more apparent in the treatment group due to a greater spread of values at the extremes of the scale. When the analysis was restricted to people with a narrower range of baseline values (between the 5^th^ and 95^th^ percentiles of the distribution [2.5 and 4 respectively]: control group n = 35; treatment group n = 33), this eliminated the interaction (t = −1.44, p = 0.156) and there was no difference in SSS symptom severity at eight weeks between groups [mean control group −0.16, treatment group −0.03; baseline-adjusted mean between-group difference (95% CI) 0.11 (−0.13, 0.34), t = 0.91, p = 0.366). If the assumption is accepted that group differences in score changes are simply due to regression to the mean, these outcomes indicate that the exercise treatment provided no benefit over advice and education alone. Neither the sensitivity analysis controlling for age, sex and disease duration nor the per protocol analyses showed substantively different results (data not shown). Adjusting the imputation to reflect the best-case scenario under a situation in which the data were not missing at random, i.e. all missing values in the treatment arm set to zero and all missing values in the control arm set to the maximum possible score, did not yield a statistically significant difference between the groups in the primary analysis [adjusted mean difference −0.39 (−0.81, 0.03), t = −1.86, p = 0.066].

### Secondary outcomes

No substantive or statistically significant differences between treatment groups were identified for the secondary or exploratory outcomes at eight weeks or 12 months, with the possible exception of walking tolerance (number of shuttles) at eight weeks (Table 4; Table S2). When all subjects were included in the shuttle test linear regression analysis at week 8 the residuals were not normally distributed. When two subjects who were clinical outliers were excluded, (one control subject with an existing foot problem at baseline whose test performance had improved dramatically at eight weeks, another randomised to the active treatment arm whose performance had dramatically declined without a corresponding increase in pain or function), the distribution of the residuals improved and the between-group difference increased. Repeating the comparison with quantile regression, which is robust to outliers and does not require residuals to be normally distributed, allowed all subjects to be included and yielded a difference of similar magnitude [adjusted median difference 6.44 (0.33, 12.55), t = 2.11, p = 0.039]. However, following correction for multiplicity this was not statistically significant. Adjusting for the additional variables, including HADS, did not affect our conclusions regarding the other secondary outcomes.

**Table 4:** Changes in secondary and exploratory outcomes at eight weeks and 12 months – unadjusted values and baseline-adjusted results.

Outcome	Mean change		Mean (95% CI) difference between groups		Linear regression
	Control (n = 38)	Active (n = 38)	Unadjusted	Adjusted	
**At 8 weeks**					
**SSS physical function** *	−0.11	−0.08	0.03 (−0.18, 0.23)	0.01 (−0.19, 0.22)	t = 0.15, p = 0.882
**N shuttles completed** **	−0.12	3.31	3.44 (−4.20, 11.07)	1.27 (−6.76, 9.31)	t = 0.32, p = 0.751
**N shuttles completed** #(excluding 2 outliers)	−1.34	4.43	5.77 (−1.32, 12.86)	4.14 (−3.63, 11.91)	t = 1.08, p = 0.287
**Oswestry**	−1.00	−0.14	0.86 (−3.07, 4.79)	0.56 (−3.35, 4.47)	t = 0.29, p = 0.776
**General Well-Being Index**	0.16	−0.08	−0.24 (−6.42, 5.94)	0.38 (−5.32, 6.08)	t = 0.13, p = 0.893
**Back pain VAS**	−11.44	−1.91	9.52 (−5.37, 24.42)	5.12 (−7.94, 18.19)	t = 0.78, p = 0.436
**Leg pain VAS**	−9.58	−4.85	4.73 (−9.90, 19.36)	3.58 (−10.42, 17.58)	t = 0.51, p = 0.609
**At 12 months**					
**SSS symptom severity**	−0.43	−0.08	0.35 (−0.06, 0.75)	0.31 (−0.07, 0.71)	t = 1.67, p = 0.102
**SSS physical function** *	−0.20	−0.07	0.13 (−0.21, 0.46)	0.11 (−0.22, 0.44)	t = 0.69, p = 0.491
**Oswestry**	−3.77	−1.27	2.50 (−5.35, 10.36)	2.19 (−5.56, 9.95)	t = 0.57, p = 0.572
**General Well-Being Index**	1.88	−1.09	−2.97 (−12.53, 6.58)	−2.26 (−11.35, 6.84)	t = –0.51, p = 0.614
**Back pain VAS**	−12.40	−0.88	11.52 (−7.39, 30.43)	7.27 (−10.28, 24.82)	t = 0.84, p = 0.407
**Leg pain VAS**	−18.63	−3.79	14.84 (−7.96, 37.63)	12.83 (−8.42, 34.08)	t = 1.24, p = 0.226

*Values are mean (95% CI) unless otherwise stated.*

* *Residuals show slight deviations from normal.*

** *Residuals not normally distributed.*

# *Residuals normally distributed.*

### Testing for unobservable selection bias

Despite finding no evidence of observable selection bias in the baseline measurements, it is still possible that unobservable selection bias could affect the outcome. When the probability of assignment to the treatment group was included as a covariate in the primary analysis this did not affect the magnitude of the between-group difference [0.03 (−0.27, 0.33), t = 0.21, p = 0.831] and P(E) was not substantively associated with the outcome [0.05 (−0.45, 0.54), t = 0.19, p = 0.849]. We similarly found no evidence of selection bias in the secondary outcomes (data not shown).

To further investigate whether patients in our sample with relatively higher symptom severity scores than others may have responded to the intervention, we repeated the secondary analyses in a subgroup of patients with baseline symptom severity scores at or above the 75^th^ percentile (control n = 10, treatment group n = 11). Whilst the mean changes were consistently in favour of the treatment group at both time-points with the exception of back pain VAS at week 8, the between-group differences were modest (Table 5).

**Table 5:** Changes in secondary and exploratory outcomes in a subgroup of patients with symptom severity scores exceeding the 75^th^ percentile at baseline.

Outcome	Mean change		Mean (90% CI) difference between groups	
	Control (n = 10)	Active (n = 11)	Unadjusted	Adjusted
**At 8 weeks**				
**SSS physical function**	0.00	−0.23	−0.24 (−0.52, 0.05)	−0.25 (−0.54, 0.05)
**N shuttles completed**	−0.11	5.88	5.99 (−9.59, 21.56)	5.03 (−9.07, 19.14)
**Oswestry**	1.09	−2.53	−3.62 (−10.59, 3.34)	−4.48 (−9.84, 0.88)
**General Well-Being Index**	0.44	−4.59	−5.03 (−17.10, 7.04)	−2.73 (−14.07, 8.61)
**Back pain VAS**	−22.45	−1.55	20.90 (−8.68, 50.49)	8.50 (−23.34, 40.34)
**Leg pain VAS**	−12.12	−21.00	−8.89 (−36.21, 18.45)	−8.95 (−36.86, 18.97)
**At 12 months**				
**SSS symptom severity**	−0.67	−0.72	−0.06 (−0.85, 0.73)	−0.12 (−0.78, 0.75)
**SSS physical function**	−0.20	−0.47	−0.27 (−1.00, 0.46)	−0.30 (−1.04, 0.45)
**Oswestry**	−4.60	−10.94	−6.35 (−23.39, 10.70)	−6.98 (−23.89, 9.93)
**General Well-Being Index**	1.55	−13.83	−15.38 (−37.83, 7.07)	−11.44 (−33.37, 10.49)
**Back pain VAS**	−16.65	−4.28	12.36 (−35.42, 60.14)	−9.52 (−59.74, 40.70)
**Leg pain VAS**	−16.63	−15.08	1.55 (−50.40, 53.50)	−2.19 (−47.30, 51.67)

No adverse events were reported by any participants during the trial period.

## Discussion

The results of this trial indicate that the self-directed programme of flexion and aerobic type exercises delivered in this study did not systematically improve symptom severity in a typical group of primary care-based NHS patients, either in the short term or long term. For the primary outcome of SSS symptom severity at eight weeks, there was no substantive difference between the two groups in the majority of the study population. There was, however, some evidence that for those with higher baseline scores for symptom severity, the physiotherapy exercise programme resulted in an improvement in SSS scores at 8 week follow-up (Table 3). This may have been due to regression to the mean; excluding patients with baseline scores at the extremes of the distribution served to eliminate the interaction and no substantive treatment effect was identified in the remaining patients. In the patients whose baseline symptom severity scores were at or above the 75^th^ percentile, mean improvement in all secondary outcomes at eight weeks except the back pain VAS was greater in the exercise treatment subgroup than the control subgroup, although the differences were modest (see Table 4). These unplanned exploratory analyses might indicate that an exercise programme may have a beneficial effect in patients with more severe symptoms, although further trials would be needed to explore this explicitly.

### Limitations of study

Because baseline symptom severity score was found to interact with the magnitude and direction of the treatment effect, assessment of the efficacy of the intervention was complicated. Reasons for the interaction were, however, explored using unplanned subgroup analyses and estimates of the treatment effect for a variety of baseline scores were calculated to aid interpretation. The use of block randomisation could conceivably have allowed the allocation concealment to be subverted, however we found no evidence that this had happened. A further criticism could be that the effect of greater physiotherapy contact in the active treatment group was not controlled for. However, the design of this trial is pragmatic, and it is therefore accepted that part of any therapeutic effect in the exercise group might be derived from the greater therapist contact and support [26].

### Results in context

Up to six contact appointments for each patient in the intervention group were allowed for in the trial protocol, but in practice adhesion to the protocol appointment schedule was poor. Patients receiving the exercise programme treatment in fact received a mean of just three treatment appointments, reflecting current pressures on NHS clinicians to minimise treatment contact times. Whilst designed as a pragmatic study, this low level of supervision for the home exercise programme may have adversely affected the trial outcomes. Providing some insight into the importance of intensity of intervention, one recently published trial [26] used a similar exercise programme, but delivered it as an intensive and supervised intervention; participants in the exercise groups attended a rehabilitation department to carry out exercises 5 days a week over a 3 week period. This trial reported that Oswestry Disability Index scores, measured treadmill walking tolerance, and visual analogue scores for back and leg pain all improved significantly in two groups receiving this intensive exercise therapy protocol compared to a control group. Although the trial was limited by its smaller sample size (n = 45), these results might suggest that in order to be effective, an exercise programme may need to be intensive and supervised. As has been shown in other degenerative conditions [27], the current trend of reducing physiotherapy contact times in the NHS in response to competition and cost-cutting pressures may reduce the resulting benefit of otherwise potentially effective physiotherapy treatments to below efficacious levels.

## Conclusions

The home exercise programme in this trial, which was based on current clinical physiotherapy practice, did not yield any systematic improvement in symptoms or function. Exploratory subgroup analysis suggested that exercises may be beneficial in patients with more severe symptoms, although this may simply represent a regression to the mean. Therefore, the effectiveness in different subgroups requires further direct evaluation.

### Clinical implications

Based on the results of this trial, an outpatient prescribed, home exercise programme cannot be recommended as an effective treatment for neurogenic claudication, especially in mild to moderate cases. Education and advice on self-management may be adequate for NC patients with mild symptoms, as there was no deterioration observed over time in the control group. For more severely symptomatic patients, future research should include an evaluation of whether an exercise programme needs to be intensive and supervised in order to produce clinical benefits before surgical interventions are considered.

## Supporting Information

Checklist S1 (DOC)

Protocol S1 **Protocol for trial.**
(DOC)

Appendix S1 **Advice and education information sheet.**
(DOC)

Appendix S2 **Exercise sheet.**
(DOC)

Table S1 **Baseline characteristics of patients for whom P(E)≠0.5.**
(DOCX)

Table S2 **Changes in primary, secondary and exploratory outcomes at eight weeks and 12 months.**
(DOCX)
