# Supplementary material for: A Home Exercise Programme Is No More Beneficial than Advice and Education for People with Neurogenic Claudication: Results from a Randomised Controlled Trial
Source: PLoS One. 2013 Sep 30;8(9):e72878. doi: 10.1371/journal.pone.0072878 (PMC3787048; doi:10.1371/journal.pone.0072878)
Supplement: Protocol S1 — Protocol for trial. (DOC) [file pone.0072878.s002.doc]

**STUDY PROTOCOL REC Ref: 08/H1313/1 C Comer**

**Condition-specific physiotherapy for neurogenic claudication:**

**a randomised trial of older adults in a primary care based physiotherapy service**

**Background Information**

*Chronic low back pain is common in older adults:*

Chronic back pain is characteristic in older people^(1)^, with 40% of those with back pain aged 65 or over reporting restriction in activity. Lumbar spinal stenosis is a common back condition in older patients, and the associated symptoms of neurogenic claudication can cause significant disability.

*Neurogenic claudication: a clinically meaningful subgroup of chronic low back pain:*

Neurogenic claudication (NC) is a clinical presentation offering an identifiable subgroup of low back pain patients. The terms lumbar spinal stenosis (LSS) and neurogenic claudication are often used interchangeably, although NC is a clinical syndrome and LSS is a pathoanatomical finding referring to narrowing of the spinal or nerve root canals and diagnosed by CT or MRI imaging. Narrowing can be caused by age-related degeneration^(2)^, but as LSS is not always symptomatic^(3)^ the term 'spinal stenosis' may have little clinical meaning^(4)^. In the non-surgical setting, the syndrome of NC provides a recognisable and meaningful clinical subgroup of chronic low back pain patients, defined by posture-related symptoms without the need for expensive spinal imaging.

*What is known about Neurogenic Claudication?*

NC has been described as 'poorly localized pain, paraesthesiae and cramping of the lower extremities of a neurologic origin'^(5)^. Onset of NC symptoms is usually insidious, developing over time following a history of chronic low back pain. These symptoms are posture-related, associated with walking and standing^(2)^, when the cauda equina/lumbosacral nerve roots and their blood supply are compressed^(5,6,7,8)^ and walking tolerance can become severely limited^(5,9,10,11)^.

*Current management of Neurogenic Claudication:*

There are no data on treating NC as a clinical syndrome without the additional use of diagnostic imaging to confirm the presence of LSS. This is despite acknowledgement in the literature that imaging findings are costly to acquire and correlate poorly with clinical presentation^(4)^. Thus, there is a substantial literature related to the management of LSS, rather than NC, and this mostly examines surgical therapies.

Surgical treatment aims to alleviate compression on the neural tissues. There is, however, 'considerable variation in long-term benefit from surgical decompression'^(6)^, and a recent Cochrane review concludes that data on indications for surgery and predictors of surgical outcome are lacking^(12)^. Surgery is not, therefore, always the treatment of choice for patients with LSS-related NC. Non-surgical interventions, including physiotherapy are recommended almost universally for initial treatment^(6,13,14,15)^. However, there is no consensus on what this non-surgical treatment should be.

Physiotherapy: There is evidence to support the use of exercise therapy for chronic back pain conditions in general^(16/17)^, but it is increasingly recognised that research is required to investigate specific treatments and exercise programmes for defined subgroups of low back pain^(18,19,20)^. The few non-RCT studies of exercise therapy for patients with LSS and NC^(21-25)^ suggest that condition-specific exercises may be beneficial. A thorough review of the literature, however, has revealed no randomised trials evaluating the effectiveness of specific exercise therapy for NC compared to natural history.

*Why conduct this trial in Leeds?*

The Leeds Musculoskeletal (MSK) Service is a city-wide, primary-care run service which sees 28,000 referrals per year. A recent audit indicated that one third of referrals were for spinal problems, of which three quarters were lumbar spine problems. The MSK service has close links with the Leeds neurosurgical service which provides a tertiary spinal surgery service to the population of Yorkshire with least 8 patients per month listed for surgery for LSS.

The proposed principal investigator holds both a clinical NHS contract with the Leeds PCT and a University academic contract. The collaboration between the primary care MSK service and experienced trialists within the Academic Unit of Musculoskeletal Disease will combine extensive clinical experience with expertise in clinical trial design and analysis. The collaboration will also help develop further the clinical research culture among allied health professionals working in the NHS team.

**Summary of Background**

NC is a common, clinically-definable subset of older low back pain patients, which does not require MRI for diagnosis. Physiotherapy is generally recommended as first line therapy, but clinical trial evidence to support this recommendation is lacking. Given the posture-dependent nature of NC symptoms, we hypothesise that a rehabilitation program focussed on posture would be a rational intervention. The conservative management of NC has been identified as a research priority need by the Agency for Healthcare Research and Quality^(13)^.

**Aims and purpose of the proposed research**

The aim of the study is to establish the effectiveness of an exercise programme aimed at addressing the specific posture-related symptoms of this condition. The purpose of the trial is to investigate the null hypothesis that a tailored exercise programme is no more effective than simple advice in improving symptoms and function in older patients with neurogenic claudication.

*Primary objective*

■ To investigate whether a condition-orientated physiotherapy exercise programme is more effective than education and advice in reducing pain and disability in the short-term.

*Secondary objectives*

■ To assess changes in subjectively reported disability and general health status

■ To investigate changes in objective measurements of walking tolerance

■ To ascertain if any improvement in either group is maintained at 12 month follow-up

■ To assess further healthcare use during a 12 month follow-up period

**Trial procedure**

This is a single blind pragmatic randomised controlled trial. Both the intervention group and the control group will receive educational information and advice. Subjects randomised to receive the active physiotherapy intervention in addition to advice will be taught a rehabilitation programme tailored to address the posture-related pain and dysfunction associated with neurogenic claudication.

*Outcome Measurement*

Primary outcome measures will consist of score changes in the symptom severity scale of the Swiss Spinal Stenosis (SSS) questionnaire to be measured at 8 week follow-up assessment. The SSS has been been used since the early 1990s, and was specifically designed for the evaluation of patients with symptoms associated with lumbar spinal stenosis. It has been shown to be a reliable and valid outcome measure for this patient group^(26)^.

Secondary outcome measures will include the Oswestry Disability Index (a commonly used measure of pain and function in low back pain), the General Well-being Index (providing a quality of life measure), objective measurement of walking tolerance (shuttle walking test), and 12 month Swiss Spinal Stenosis score.

*Sample size*

This study will be powered to detect a 0.5 point difference in the primary outcome measure; the Swiss Spinal Stenosis questionnaire symptom severity scale: The Minimum Clinically Important Difference for scores in this measure has been determined previously as a change of at least 0.5 points in the symptom severity scale and in the physical function scale ^(27)^. Population standard deviations of samples reported in previous studies ^(28,29)^ range from 0.48 to 0.56; and the more conservative figure of 0.56 has been adopted for powering this study.

The proposed sample size is based on the ability to detect a difference (Δ) in treatment groups of 0.5 points in the symptom severity scale, with a standard deviation of 0.56, and based on a power of 90% (alpha 5%). The required sample size at exit is 28 subjects and 28 controls. To allow for group size differences due to randomisation, and for a drop-out rate of 20%, a total recruitment of 76 patients is planned.

*Subject Recruitment*

Subjects will be recruited from patient referrals to the Leeds primary care Musculoskeletal service or Neurosurgical service. The Leeds Musculoskeletal (MSK) Service is a city-wide, primary-care run service which sees approximately 28000 referrals per year. A recent audit of the primary care MSK service reported that almost one third of referrals were for spinal problems, of which three quarters were lumbar spine problems. The number of lumbar spine referrals over the recruitment time of 18 months can therefore be conservatively estimated to be approximately 10,400. Using previously published prevalence figures which suggest that 3-4% patients with low back pain are older patients with lumbar spinal stenosis, and based on a conservative 50% prevalence of neurogenic claudication amongst this group, approximately 200 referrals for patients with neurogenic claudication over 18 months through the primary care MSK service can be estimated. In addition, patients with neurogenic claudication will be recruited from referrals to the Leeds neurosurgical service which provides a tertiary spinal surgery service to the population of Yorkshire and where currently at least 8 patients per month are listed for surgery for LSS.

Potentially eligible patients will be sent a study information leaflet providing details of the aims of the study and what participation in the trial will involve. Subjects will subsequently be contacted by telephone for initial screening and to ascertain willingness to participate. Interested eligible subjects will then attend a clinic appointment for final screening for inclusion and exclusion criteria. Eligible subjects who are willing to participate will be recruited by the study co-ordinator after informed consent has been gained.

Inclusion criteria

1. Age 50 years or older with symptoms of NC, defined as bilateral non-dermatomal lower limb symptoms (below the gluteal fold), induced on erect walking and eased in sitting or flexion of the lumbar spine. Lower limb symptoms may include pain, heaviness, aching, cramping, paraesthesia, or weakness.

2. Limitation of normal walking tolerance or function due to symptoms of neurogenic claudication

Exclusion criteria will include subjects with clearly defined radicular symptoms (sciatica), cognitive impairment or medical conditions preventing understanding or participation in the study, and subjects with symptoms requiring urgent surgical or other intervention (red flags – see below).

Red Flags (CSAG 1994) – Indicators of potential serious pathology

■ Age of onset <20 or > 55 years

■ Violent trauma, eg fall from a height, road traffic accident

■ Constant progressive, non-mechanical pain

■ Thoracic pain

■ Past medical history of carcinoma

■ Systemic steroids

■ Drug abuse, HIV

■ Systemically unwell

■ Weight loss

■ Persistent severe restriction of lumbar flexion

■ Widespread neurology

■ Structural deformity

*Randomisation*

Treatment will be determined by simple block randomisation. Web-based randomisation will occur after written informed consent and baseline measures have been obtained and will be based on a computer-generated randomisation protocol.

*Study plan*

Following recruitment to the study, participants will be randomised either to the advice only intervention, or to the advice plus active physiotherapy rehabilitation intervention. It will not be possible to blind subjects to the treatment they receive, and it is recognised that outcome is possibly influenced by preconceived ideas regarding the effectiveness of intervention^(30,31)^. Subjects' treatment preferences will therefore be elicited prior to randomisation, so that this information may be used to inform the analysis of outcomes.

**Common Intervention to both treatment groups: Education and advice**

All subjects will receive verbal and written advice and education on lumbar spinal stenosis based on a patient information publication on spinal stenosis written by the American College of Rheumatology^(32)^ emphasising the benefits of remaining active. (There are currently no guidelines or 'standard care' relating to conservative treatment for NC or LSS or directed at older patients with degenerative spinal conditions, but European Guidelines for the management of chronic non-specific low back pain with or without referred leg pain recommend brief educational interventions among other recommended treatments.)

**Physiotherapy exercise programme**

Each patient randomised to this group will be taught a programme of exercises by the treating physiotherapist, selected from a list of possible exercises (see below) and will be instructed to perform the selected programme of exercises twice daily. These exercises are based on flexion movements and postural adaptation to reduce symptoms from neural compression and include exercises improve fitness. The treating physiotherapist will review each subject at least 4 times and up to six times during the 6 week treatment period and will guide each subject in progressing the exercises (intensity, duration or repetitions) as appropriate. The physiotherapist will encourage compliance during treatment and encourage continuation of the exercise programme after the treatment period. In order to standardise the physiotherapy rehabilitation programme, a training session for physiotherapists administering interventions will be held by the study co-ordinator prior to commencement of the study.

*Follow-up*

All outcome measures will be repeated at 8 weeks and questionnaire outcomes will be repeated at 12 months from randomisation. These measures will be administered by the study co-ordinator who will be blinded to the intervention received.

All clinical assessment and treatment sessions will be administered in primary care outpatient clinics most convenient to the study participants.

*Dropouts and anticipated problems with compliance*

Problems with compliance and participant dropout from the trial are expected to be minimal due to the nature of the interventions and relatively short trial period. For genuine dropouts, every effort will be made to retrieve exit data for the primary endpoint, which may include telephone follow-up. The intention-to-treat approach to the analysis of this pragmatic trial designs ensures that compliance failures in both treatment arms will contribute valid data to the evaluation.

*Statistical Analysis*

The Academic Unit employs a statistician who has input into preparation of this application and who will work with the study team during the analysis phase. Descriptive exploration will compare the pre and post intervention data for the two groups, exploring for change in Swiss Spinal Stenosis (SSS) symptom severity and physical function scores. Inferential analysis of the pre-and post-intervention SSS symptom severity data will be based on multi-variable models appropriate to the data type, including baseline data as covariates (ANCOVA). The primary analysis will be undertaken according to intention to treat principles, with missing data excluded. A secondary analysis will also be performed where missing data will be analysed assuming worst-case values.

*Study timetable*

The study will have a 3 month run-in period, and will allow 18 months for recruitment.

Following 12 month follow-up, a further 3 months will be allowed for data cleansing, analysis, and preparation of the final report by the end of the three year trial period.

*Trial Management*

A trial steering committee, with an independent chair and lay representation will be set-up within the Leeds Academic Unit of Musculoskeletal Disease. The trial steering committee will receive 3-monthly reports from the study co-ordinator regarding recruitment and trial progress and will be responsible for external reporting to the funder and regulatory authorities. This committee, in conjunction with the Academic Unit's standing data-monitoring committee (chaired by Professor HA Bird) will monitor adverse event management and all data-related aspects of the management of the trial. Day to day trial management will be overseen by a trial management group comprising the grant holders, the department's business manager and trial staff.

**Advice and Information**

**Verbal education and advice**

Standardised verbal education and advice will be provided for all study participants at their first physiotherapy clinic appointment. This information will include:

- Description of Neurogenic claudication symptoms

- Description of spinal stenosis and the pathological process causing symtpoms

- Explanation of the diagnosis, based predominantly on medical history

- Brief explanation of the causes of stenosis, predominantly degenerative changes in the

lumbar spine

- Explanation of how and why posture affects the symptoms of neurogenic claudication

**- Discussion of the available treatments for lumbar spinal stenosis, including**

**physiotherapy, spinal injection, surgery.**

**- Discussion around the benefits of exercise, and improving/maintaining fitness**

**Written education and advice**

**A written information and advice leaflet will be given to all study participants on their first physiotherapy clinic appointment. This leaflet is based on information from the American College of Rheumatology patient information website.**

**Exercise programme**

*The following exercise programme is aimed at addressing the specific dysfunctions associated with neurogenic claudication and is based on exercises advocated in current available literature and research in this area.*

*Your physiotherapist should prescribe at least one exercise selected from each section*

*(1 – 5) of the exercise rehabilitation programme outlined below. In addition, stretching exercises may be given as appropriate. You should aim to do your exercises twice daily.*

**1) LORDOSIS MINIMISING POSTURAL EXERCISES**

*progress from a. to e. as appropriate*

a. Posterior pelvic tilt/lordosis flattening exercises in standing against wall, knees slightly

flexed

b. Posterior pelvic tilt/lordosis flattening exercises in standing against wall with knees

extended

c. Posterior pelvic tilt/lordosis flattening exercises in standing without wall

d. Posterior pelvic tilt/lordosis flattening exercises while marching on spot

e. Posterior pelvic tilt/lordosis flattening exercises during normal walking

**2) SPINAL FLEXION EXERCISES**

*include one of the following exercises*

a. Knee to chest curls in crook lying

b. Lumbar flexion in sitting – reaching fingertips to floor

c. Lumbar flexion exercises in standing - reaching fingertips to ankles

**3) ABDOMINAL MUSCLE STRENGTHENING EXERCISES**

*include one of the following exercises*

a. static transverse abdominus muscle static contraction in crook lying

b. alternate knee lifts in crook lying, maintaining neutral lumbar position

c. abdominal curls reaching finger tips to knees

**4) GLUTEAL/ TRUNK MUSCLE STRENGTHENING EXERCISES**

*include one of the following*

a. bridging, weightbearing through both feet

b. bridging, one leg crossed over the other, weightbearing through one foot only

c. bridging, extending alternate knees

**5) AEROBIC CONDITIONING EXERCISES**

*include one of the following exercises to tolerance, increasing by 1 minute*

*each week*

a. knee dip/ calf raise alternating exercise

b. walking

c. cycling

d. step-ups

**6) STRETCHES** *include if required*

a. hip flexor muscle stretches (in prone lying, side lying or standing)

b. calf muscles stretches (in standing or on step)

**References**

1.The prevalence of back pain in Great Britain in 1998: London: Bulletin 1999/18 Government Statistical Service Bulletin 1999

2.Goh KJ, Khalifa W, Anslow P, Cadoux-Hudson T, Donaghy M. The clinical syndrome associated with lumbar spinal stenosis. European Neurology 2004; 52(4): 242-249

3.Haig AJ, Tong HC, Yamakawa KS, Quint DJ, Hoff JT, Chiodo A et al. Spinal Stenosis, Back pain, or no symptoms at all? A masked study comparing radiologic and electrodiagnostic diagnoses to the clinical impression. Arch Phys Med Rehabil 2006. 87; 897-903

4.Haig AJ, Tong HC, Yamakawa KSJ, Parres C, Quint DJ, Chiodo A, Miner JA et al. Predictors of Pain and Function in Persons with Spinal Stenosis, Low Back Pain and No Back Pain. Spine 2006; 31(25): 2950-2957

5.Porter RW. Spinal Stenosis and Neurogenic Claudication. Spine 1996: 21(17): 2046-2052

6.Atlas SJ & Delitto A. Spine Stenosis; Surgical versus Nonsurgical Treatment. Clinical Orthopaedics and Related Research 2006: 443; 198-207

7.Bal S, Celiker R, Palaoglu S, Cila A. F wave studies of neurogenic intermittent claudication in lumbar spinal stenosis. Am Journal of Physical Medicine and Rehabilitation 2006: 85(2); 135-140

8.Garfin SR, Herkowitz HN, Mirkovic S. Spinal stenosis. J Bone Joint Surgery 1999: 81; 572-586

9.Amundsen T, Weber H, Lilleas F, Nordal H, Abdelnoor M, Magnaes B. Lumbar spinal stenosis: clinical and radiologic features. Spine 1995: 20(10); 1178-1186

10.Katz JN, Lipson SJ, Brick GW, Grobler LJ, Weinstein JN, Fossel AH, Liang MJ. Clinical correlates with patient satisfaction after laminectomy for degenerative lumbar spinal stenosis. Spine 1995; 20:1155-1160

11.Stucki G, Liang MH, Lipson SJ, Fossel AH, Katz JN. Contribution of neuromuscular impairment to physical function in patients with lumbar spinal stenosis. J Rheumatol 1994; 21: 1338-1343

12.Gibson JNA, Waddell G. Surgery for degenerative lumbar spondylosis. Cochrane Database of Systematic Reviews 2005, Issue 4. Art. No: CD001352. DOI: 10.1002/14651858. CD001352.pub3

13.Agency for Healthcare Research and Quality. Treatment of Degenerative Lumbar Spinal Stenosis. Summary, Evidence Report/Technology Assessment 2001: Number 32. AHRQ Publication No 01-E047, March 2001 http://www.ahrq.bov/clinic/epcsums/stenosum.htm

14.Amundsen T, Weber H, Nordal H, Magnaes B, Abdelnoor M, Lilleas F. Lumbar Spinal Stenosis: Conservative or Surgical Management? A Prospective 10-year Study. Spine 2000; 25(11) 1424-1436

15.Simotas AC, Dorey FJ, Hansraj KK, Cammisa F. Nonoperative Treatment for Lumbar Spinal Stenosis Clinical and Outcome Results and a 3 Year Survivorship Analysis. Spine 2000; 25 (2) 197-204

16.Hayden JA, van Tulder MW, Malmivaara A, Koes BW. Exercise therapy for treatment of non-specific low back pain. Cochrane Database of Systematic Reviews 2005, Issue 3. Art. No: CD000335. DOI: 10.1002/14651858. CD000335.pub2

17.Araiksinen O, Hildebradt J, Mannion AJ, Ursin H, Brox JI, Klaber-Moffett J et al: European Guidelines for the Management of Chronic Non-specific Low Back Pain 2004

18.O'Sullivan P Diagnosis and classification of chronic low back pain disorders: maladaptive movement and motor control impairments as underlying mechanism. Manual Therapy 2005: 10; 242-255

19.Fritz JM, Delitto A, Erhard RE. Comparison of classification-based physical thearpy with therapy based on clinical practice guidelines for patients with acute low back pain. Spine 2003: 28(13); 1363-1372

20.Brennan GP, Fritz JM, Hunter SJ, Thackeray A, Delitto A, Erhard RE. Identifying subgroups of patients with acute/subacute 'nonspecific' low back pain. Results of a randomized clinical trial. Spine 2006: 31(6); 623-631

21.Houedakor J, Cabre P, Pascal-Moussellard H, Gallien P, Rene-Corail P, Smadja D. Rehabilitation treatment in lumbar stenosis. Preliminary results of a prospective study. Annales Readaptation Medecine Physique 2003; 46: 227-232

22.Zeifang F, Abel R, Schiltenwolf M. Possible conservative treatment methods for patients with spinal claudication. Der Orthopade 2003: 32; 906-910

23.Simotas AC. Nonoperative Treatment for Lumbar Spinal Stenosis. Clinical Orthopaedics and Related Research 2001: 384; 153-161

24.Fritz JM, Erhard RE, Vignovic M. A nonsurgical treatment approach for patients with lumbar spinal stenosis. Physical Therapy 1997: 77(9); 962-973

25.DuPriest CM. Nonoperative management of lumbar spinal stenosis. J of Manipulative and Phsysiological Therapeutics 1993: 16(6); 411-414

26.Pratt R K, Fairbank JCT, Virr A. The Reliability of the Shuttle Walking Test, the Swiss Spinal Stenosis Questionnaire, the Oxford Spinal Stenosis Score, and the Oswestry Disability Index in the Assessment of Patients with Lumbar Spinal Stenosis. Spine 2002; 27(1): 84-91

27.Tuli SK, Yerby SA, Katz JN. Methodological Approaches to Developing Criteria for Improvement in Lumbar Spinal Stenosis Surgery. Spine 2006; 31(11): 1276-1280

28.Zucherman JF, Hsu KY, Hartjen CA et al (2005): A Multicenter, Prospective, Randomized Trial evaluating the XSTOP Interspinous Process Decompression system for the treatment of Neurogenic Intermittent claudication. Two year follow-up results. Spine 30(12): 1351-1358

29.Whitman JM, Flynn TW, Childs JD et al (2006): A comparison between two physical therapy treatment programs for patients with lumbar spinal stenosis. A randomized clinical trial. Spine 31(22): 2541-2549

30.Verbeek J, Sengers MJ, Riemens L, Haafkens J. Patient expectations of treatment for back pain: a systematic review of qualitative and quantitative studies. Spine 2004; 29: 2309-2318

31.Klaber-Moffett J, Torgerson D, Bell-Syer S, Jackson D, Llewlyn-Phillips H, Farrin A, Barber J. Randomised controlled trial of exercise for low back pain: clinical outcomes, costs, and preferences. BMJ 1999. 319: 279-283

32.Sarzi-Puttini Pc, Mody E. American College of Rheumatology Patient Education Task Force. Spinal Stenosis. .http://www.rheumatology.org/public/factsheets/stenosis.asp 2006
