# Supplementary material for: A Home Exercise Programme Is No More Beneficial than Advice and Education for People with Neurogenic Claudication: Results from a Randomised Controlled Trial
Source: PLoS One. 2013 Sep 30;8(9):e72878. doi: 10.1371/journal.pone.0072878 (PMC3787048; doi:10.1371/journal.pone.0072878)
Supplement: Appendix S1 — Advice and education information sheet. (DOC) [file pone.0072878.s003.doc]

**Information leaflet**

**Neurogenic Claudication and Spinal Stenosis**

**What is Neurogenic claudication?**

Neurogenic claudication is a term used to describe the leg pain and symptoms during walking which are associated with the condition of lumbar spinal stenosis. Spinal stenosis (or narrowing) is a common condition that occurs when the small spinal canal that contains the nerve roots and spinal cord becomes restricted. This narrowing can squeeze the nerves and the spinal cord causing lower back and leg pain. In general, spinal narrowing is caused by osteoarthritis, or “wear and tear” arthritis, of the spinal column. This results in a “pinching” of the spinal cord and/or nerve roots.

**People suffering from neurogenic claudication have trouble walking any significant distance, and frequently must sit or lean over forward on a grocery cart, countertop or assistive device such as a walker. While there are no cures, there are many therapies available.**

### What is spinal stenosis
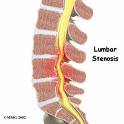
?

Spinal stenosis is a narrowing of one or more areas of the spine. This narrowing, which occurs most often in lumbar region (lower back) can put pressure on the spinal cord or nerves branching out from the compressed areas.

### ****What are the symptoms?****

**Typically, a person with spinal stenosis complains about pain in the legs or calves and lower back after walking. This is called neurogenic claudication, and the symptoms are usually relieved quickly by sitting down, or leaning over. When the spine is bent forward, more space is available for the spinal cord, causing a reduction in symptoms.**

Although symptoms may arise from narrowing of the spinal canal, not all patients with narrowing develop symptoms of neurogenic claudication. Why some patients develop symptoms and others do not remains unknown.

### What causes it?

**Unless the individual is born with a small spinal canal (congenital stenosis), spinal narrowing most commonly results from progressive degenerative changes. This “acquired spinal stenosis” can occur from the narrowing of the space around the spinal cord due to bony overgrowth from osteoarthritis combined with thickening of one of the ligaments in the back, and a bulge of the intervertebral discs.**

### ****Who gets it?****

The risk of developing spinal stenosis increases in those who:

- Are born with a narrow spinal canal
- Are female
- Are 50 years of age or older
- Have had previous injury or surgery of the spine

Conditions that can cause spinal stenosis include osteoarthritis, trauma, previous spinal surgery, tumours, and Paget's disease.

**How is it diagnosed?**

You will be asked about your symptoms and medical history, and perform a physical exam if spinal stenosis is suspected. Your symptoms may include: numbness, weakness, cramping, or pain in the legs and thighs; radiating pain down the leg; abnormal bowel and/or bladder function; decreased sensation in the feet causing difficulty placing the feet when walking; loss of sexual function; and/or partial or complete leg paralysis.

Additional tests, such as x-rays or MRI scans are sometimes used if the diagnosis is unclear.

### How is it treated?

### Although there is no cure for spinal stenosis, various therapies are available, one of the most important being exercise. Keeping the hip and leg muscles from getting weaker helps increase stability and the ability to walk.

Medications may be helpful in pain relief and cortisone injections into the epidural space, the area around the spinal cord, can give relief to some people.

Under severe circumstances, surgery to correct this disorder may be appropriate. However, adequate decompression of the nerves and maintenance of bony stability are necessary for a good surgical outcome for patients with spinal stenosis.

**Several studies report that surgical treatment produces better outcomes than non-surgical treatment in the short term. However, results tend to deteriorate with time. Lumbar decompressive surgery can be complicated by epidural hematoma, deep venous thrombosis, dural tear, infection, nerve root injury and recurrence of symptoms.**

### Living with spinal stenosis

- Exercise regularly. Regular exercise, which focuses on flexion-based exercise (bending forwards) often reduces pain symptoms. Add in some walking, swimming and stretching exercises for even better results.
- Modify activity. Avoid activities that can cause or worsen pain and disability.
- Talk to your GP about pain medications, and other methods recommended for pain reduction.
- **Explore non-surgical options first except in rare cases of rapid neurologic progression or cauda equina syndrome.**

### Fast Facts

- Neurogenic claudication describes the leg pain and symptoms which are brought on by walking caused by spinal stenosis.
- Spinal stenosis is typically the result of osteoarthritis causing a pinching of the spinal cord.

- Medical history is key in making the diagnosis of spinal stenosis.
- Anyone over the age of 50 is at risk.
- The impact of stenosis on a particular patient can vary from minimal to severe.
- Exercising regularly to keep muscles functioning can reduce discomfort and maintain strength
- **How the symptoms are affecting quality of life should direct treatment choices.**
- There are no cures for spinal stenosis, but therapies can assist in regaining mobility and comfort.
- **Exercise is of paramount importance.**

**This information leaflet is based on patient information from the American College of Rheumatology website written by P Sarzi-Puttini M.D. and E Mody M.D, and reviewed by the Rheumatology Patient Education Task Force, September 2006: www.rheumatology.org**
