# Supplementary material for: A Home Exercise Programme Is No More Beneficial than Advice and Education for People with Neurogenic Claudication: Results from a Randomised Controlled Trial
Source: PLoS One. 2013 Sep 30;8(9):e72878. doi: 10.1371/journal.pone.0072878 (PMC3787048; doi:10.1371/journal.pone.0072878)
Supplement: Appendix S2 — Exercise sheet. (DOC) [file pone.0072878.s004.doc]

**Exercise programme: Neurogenic Claudication Physiotherapy Rehabilitation study**

*Your physiotherapist should prescribe at least one exercise selected from each*

*section (1 – 5) of the exercise programme outlined below.*

***You should aim to do your exercises twice each day.***

**1) BACK FLATTENING EXERCISES**

*progress from a. to e. as appropriate*

a. Pelvic tilt / lower back flattening exercises

while standing against wall, knees

slightly bent. Hold for 20 seconds.

Repeat 3 times.

b. Pelvic tilt / lower back flattening exercises

while standing against wall with knees

straight. Hold for 20 seconds.

Repeat 3 times.

c. Pelvic tilt / lower back flattening exercises

while standing without wall. Hold

for 20 seconds. Repeat 3 times

d. Pelvic tilt / lower back flattening exercises

while marching on spot. Keep marching

for 20 seconds. Repeat 3 times.

e. Pelvic tilt / lower back flattening exercises

while walking up and down. Keep walking

for 20 seconds. Repeat 3 times.

**2) SPINAL FORWARD BENDING EXERCISES**

*include one of the following exercises*

a. Knee to chest curls in crook lying. Hug both knees

into your chest for 20 seconds. Repeat 3 times.

b. Forward bending sitting – reaching fingertips to floor.

Hold position for 20 seconds. Repeat 3 times.

c. Forward bending in standing - reaching fingertips

to ankles. Hold position for 20 seconds.

Repeat 3 times.


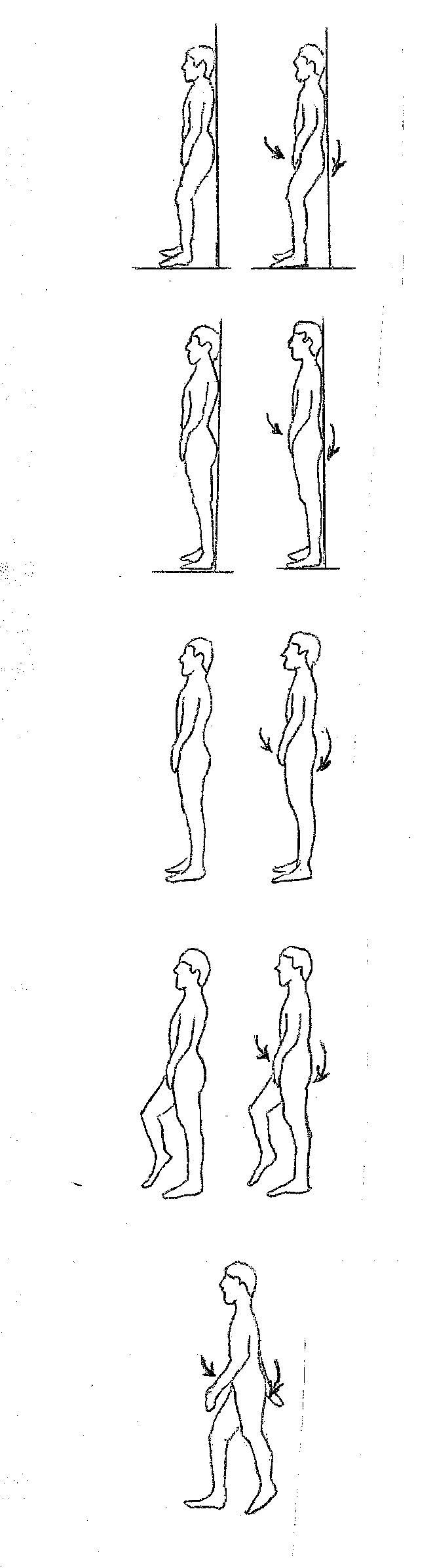


**
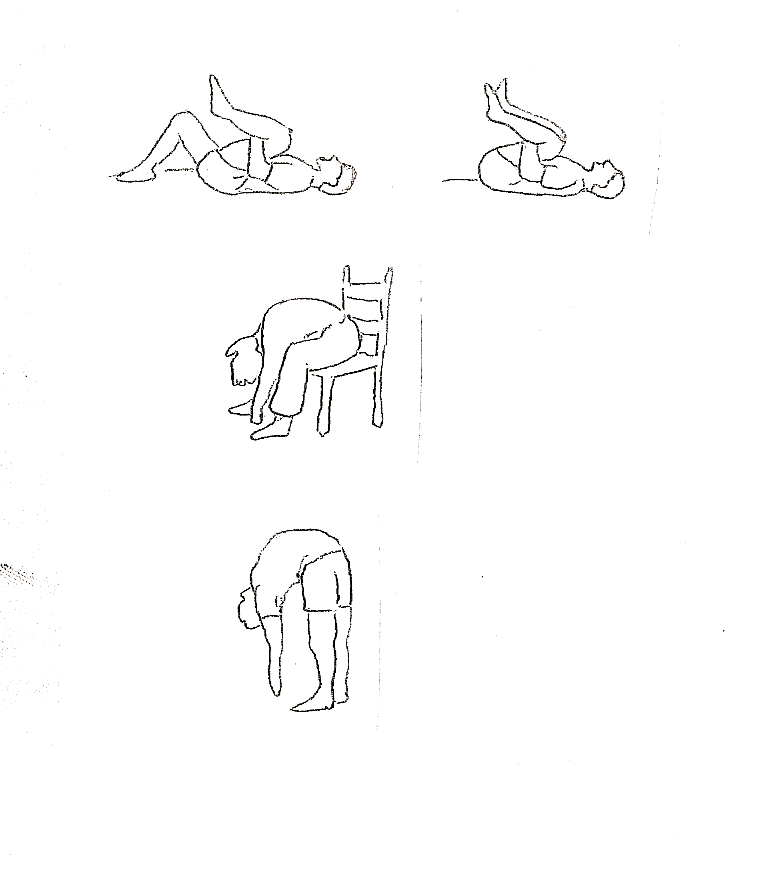
**

**3) ABDOMINAL MUSCLE STRENGTHENING EXERCISES**

*include one of the following exercises*

a. Abdominal muscle tightening.

Hold for 20 seconds. Repeat 3 times.

b. Alternate knee lifts maintaining low back position.

Hold back position steady doing 10 alternate

knee lifts. Repeat 3 times.

c. Abdominal curls reaching finger tips to knees.

Hold curled position for 20 seconds.

Repeat 3 times.

**4) GLUTEAL/ TRUNK MUSCLE STRENGTHENING EXERCISES** *include one of the following*

a. Bridging, weightbearing through both feet

Hold for 20 seconds. Repeat 3 times.

b. Bridging, one leg crossed over the other, weightbearing through one foot only

Hold for 20 seconds. Repeat 3 times on each leg.

c. Bridging, extending alternate knees

Hold bridge position while lifting each leg

alternately 3 times.

**5) AEROBIC CONDITIONING EXERCISES**

*include one of the following exercises to tolerance, increasing by 1 minute each week*

a. Knee dip/ calf raise alternating exercise

b. Brisk walking

c. Cycling

d. Step-ups

**6) STRETCHES** *include if required*

a. Hip muscles stretches (lying down or standing)

b. Calf muscles stretches (leaning against wall or on step)

*
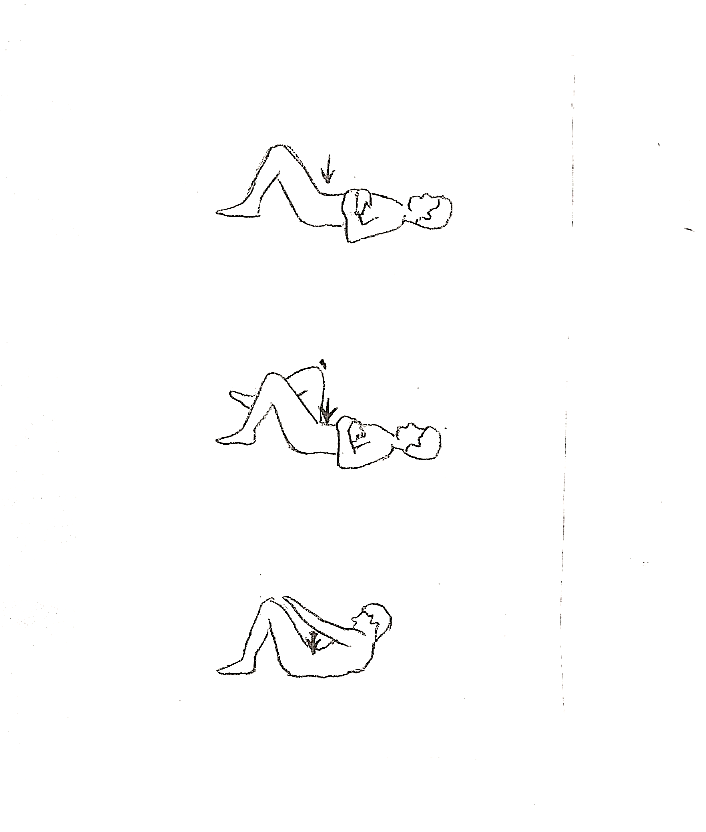
*

*
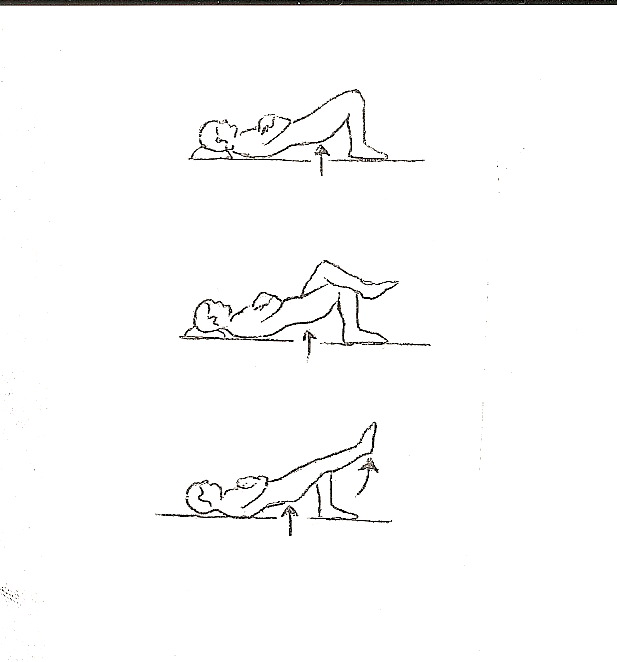
*

*
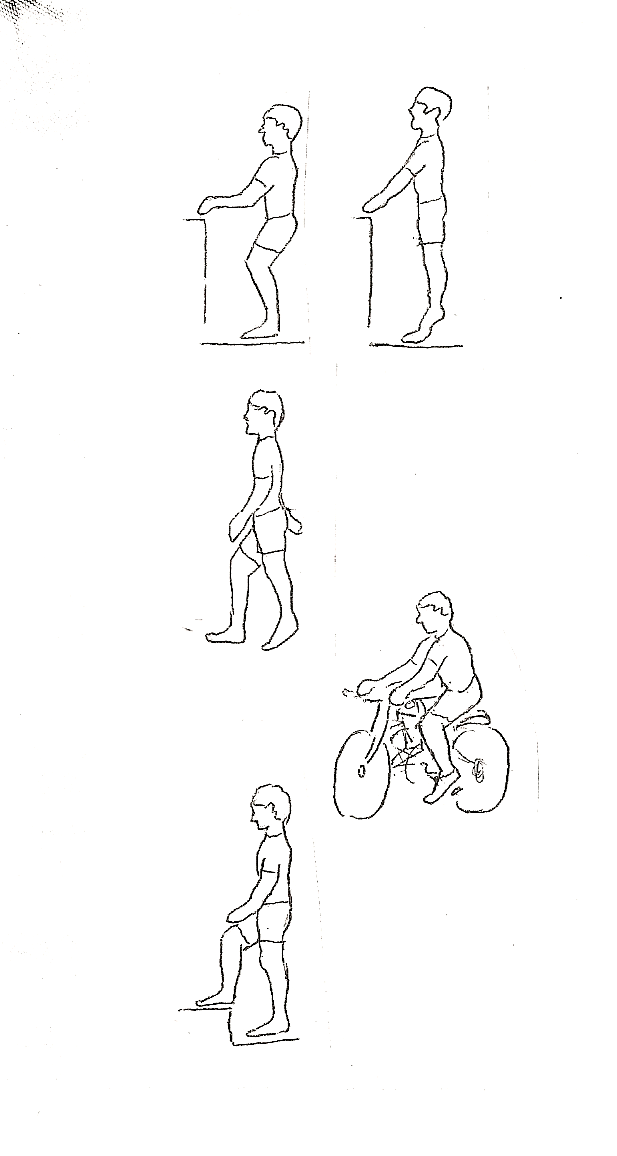
*
