# Supplementary material for: A Home Exercise Programme Is No More Beneficial than Advice and Education for People with Neurogenic Claudication: Results from a Randomised Controlled Trial
Source: PLoS One. 2013 Sep 30;8(9):e72878. doi: 10.1371/journal.pone.0072878 (PMC3787048; doi:10.1371/journal.pone.0072878)
Supplement: Table S1 — Baseline characteristics of patients for whom P(E)≠0.5. (DOCX) [file pone.0072878.s005.docx]

#### Table S1. Baseline characteristics of patients for whom P(E)≠0.5

|  | **Control n=19** | **Active n=24** |
| --- | --- | --- |
| Age, years: mean (SD), range | 69.5 (9.0), 53 to 87 | 74.7 (7.9), 61 to 86 |
| Female: n (%) | 9 (47.4%) | 13 (54.2%) |
| BMI: mean (SD), range | 27.19 (3.78), 21.41 to 24.09 (n=17) | 26.91 (3.90), 22.48 to 35.92 (n=21) |
| Duration*, years: median (IQR) | 6.0 (3.0 to 24.0) | 5.3 (0.9 to 13.8) |
| SSS symptom: mean (SD) | 3.3 (0.6) | 3.2 (0.5) |
| SSS physical: mean (SD) | 2.6 (0.3) | 2.6 (0.4) |
| Shuttles completed: median (IQR) | 21.0 (12.0 to 28.0) | 19.5 (7.0 to 27.8) |
| Oswestry score: mean (SD) | 43.7 (7.6) | 41.0 (8.1) |
| General Well-Being Index: mean (SD) | 66.7 (12.5) | 66.8 (11.8) (n=22) |
| Back pain VAS: mean (SD) | 70.6 (27.0) | 59.2 (30.6) (n=23) |
| Leg pain VAS: mean (SD) | 68.8 (22.9) | 70.4 (28.2) (n=23) |
| HADS depression: mean (SD) | 9.1 (2.9) | 9.1 (2.4) (n=23) |

*Time since onset of first symptom (back and/or leg pain)
