# Supplementary material for: A Home Exercise Programme Is No More Beneficial than Advice and Education for People with Neurogenic Claudication: Results from a Randomised Controlled Trial
Source: PLoS One. 2013 Sep 30;8(9):e72878. doi: 10.1371/journal.pone.0072878 (PMC3787048; doi:10.1371/journal.pone.0072878)
Supplement: Table S2 — Changes in primary, secondary and exploratory outcomes at eight weeks and 12 months. (DOCX) [file pone.0072878.s006.docx]

**Table S2. Changes in primary, secondary and exploratory outcomes at eight weeks and 12 months: Unadjusted values and baseline-adjusted results from quantile regression models.**

| **Outcome** | **Median change** | | **Median (95% CI) difference between groups** | | **Quantile regression** |
| --- | --- | --- | --- | --- | --- |
|  | **Control (n=38)** | **Active (n=38)** | **Unadjusted** | **Adjusted** |  |
| **At 8 weeks** |  |  |  |  |  |
| **SSS symptom severity** | -0.14 | -0.08 | 0.06 (-0.27, 0.39) | 0.01 (-0.29, 0.32) | t=0.07, p=0.941 |
| **SSS physical function** | -0.10 | -0.03 | 0.07 (-0.17, 0.30) | 0.03 (-0.16, 0.21) | t=0.29, p=0.771 |
| **N shuttles completed** | -3.70 | 3.30 | 7.00 (1.23, 12.77) | 6.44 (0.33, 12.55) | t=2.11, p=0.039 |
| **Oswestry** | -0.04 | -0.09 | -0.05 (-4.11, 4.11) | 0.21 (-3.93, 4.35) | t=0.10, p=0.920 |
| **General Well-Being Index** | 1.05 | -0.70 | -1.75 (-8.20, 4.71) | -1.27 (-8.96, 6.42) | t=-0.33, p=0.740 |
| **Back pain VAS** | -1.25 | -1.50 | -0.25 (-17.12, 16.62) | -0.36 (-19.03, 19.76) | t=0.04, p=0.970 |
| **Leg pain VAS** | -4.15 | -3.55 | -0.60 (-16.73, 17.93) | 2.16 (-14.71, 19.02) | t=0.26, p=0.799 |
| **At 12 months** |  |  |  |  |  |
| **SSS symptom severity** | -0.25 | 0.08 | 0.34 (-0.12, 0.79) | 0.28 (-0.17, 0.72) | t=1.24, p=0.219 |
| **SSS physical function** | 0.00 | 0.00 | 0.00 (-0.25, 0.25) | 0.00 (-0.24, 0.24) | t=-0.02, p=0.986 |
| **Oswestry** | -2.57 | -0.51 | 2.06 (-4.40, 8.52) | -0.53 (-6.19, 5.13) | t=-0.19, p=0.853 |
| **General Well-Being Index** | 1.64 | 2.76 | 1.12 (-6.02, 8.27) | 1.98 (-4.12, 8.09) | t=0.65, p=0.517 |
| **Back pain VAS** | -6.60 | -4.45 | 2.15 (-17.45, 21.75) | 1.78, -21.94, 25.50) | t=0.15, p=0.879 |
| **Leg pain VAS** | -13.10 | -2.25 | 10.85 (-18.55, 40.25) | 4.99 (-26.45, 36.42) | t=0.32, p=0.748 |
